# Estimation of insulin resistance in non-diabetic normotensive Saudi adults by QUICKI, HOMA-IR and modified QUICKI: A comparative study

**DOI:** 10.4103/0256-4947.65252

**Published:** 2010

**Authors:** Suhad M. Bahijri, Eman M. Alissa, Daad H. Akbar, Tawfik M. Ghabrah

**Affiliations:** a From the Department of Clinical Biochemistry, Faculty of Medicine, King Abdul Aziz University, Jeddah, Saudi Arabia; b From the Department of Medicine, Faculty of Medicine, King Abdul Aziz University, Jeddah, Saudi Arabia; c From the Department of Family and Community Medicine, Faculty of Medicine, King Abdul Aziz University, Jeddah, Saudi Arabia

## Abstract

**BACKGROUND AND OBJECTIVES:**

Identification of insulin resistance (IR) in the general population is important for developing strategies to reduce the prevalence of non-insulin-dependent diabetes mellitus (NIDDM). We used the original and a modified version of the Quantitative Insulin Sensitivity Check Index (QUICKI, M-QUICKI), and the Homeostasis Model Assessment of Insulin Resistance (HOMA-IR) to divide non-diabetic normotensive adults into high- (HIR) and low-insulin-resistant (LIR) subgroups to investigate similarities and differences in their characteristics.

**SUBJECTS AND METHODS:**

Three hundred fifty-seven healthy adults aged 18-50 years were recruited randomly from health centers in Jeddah in a cross-sectional study design. Anthropometric and demographic information was taken. Insulin, glucose, lipid profile and free fatty acid were determined in fasting blood samples. M-QUICKI, HOMA-IR and QUICKI were calculated. Reported cut-off points were used to identify HIR subjects, who were then matched for age and sex to others in the study population, resulting in 3 HIR and 3 LIR subgroups.

**RESULTS:**

Two hundred nine subjects satisfied the selection criteria. M-QUICKI correlated significantly (*P*=.01) with HOMA-IR and QUICKI values. Increased adiposity was the common characteristic of the three HIR subgroups. HIR subgroups identified using M-QUICKI (97 subjects) and HOMA (25 subjects), but not QUICKI (135 subjects), had statistically different biochemical characteristics compared to corresponding LIR sub-groups.

**CONCLUSION:**

Adiposity, but not sex, is a risk factor for IR in the studied population. Further studies are needed to choose the most appropriate index for detecting IR in community-based surveys.

Insulin resistance underlies abnormalities of glucose, lipid and blood pressure homeostasis.^1^ It is also the major factor involved in the pathogenesis of several diseases, including type 2 diabetes mellitus (T2DM), hypertension, dyslipidemias, and cardiovascular disorders.^2^ These disorders, in particular T2DM, have reached epidemic proportions in recent years.^3^ The emerging epidemics are exacerbated by the expansion of the aged population because aging facilitates aberrant insulin regulation.^4^,^5^ Furthermore, it is predicted that the aged population will increase to 32% by the year 2050, aggravating the situation further.^6^

There is an increasing prevalence of insulin-resistance-related disorders in Saudi Arabia.^7^,^8^ A recent survey stated that the prevalence of diabetes mellitus in the country has reached 23.7% in adults.^9^ Various studies have shown that insulin resistance is a strong predictive factor for the future development of the disease.^10^,^11^ Therefore, identification of insulin-resistant subjects in the general non-diabetic population is of great importance in a community-based strategy to reduce its prevalence, and hence the prevalence of non-insulin dependent diabetes mellitus (NIDDM), especially as intervention studies have demonstrated that preservation of b-cell function decreases the conversion rate of pre-diabetes to diabetes.^12^,^13^ The method of choice has to be suitable for a large-population study, should require one blood sample only, should have a high level of reproducibility and prediction power, and be easy to interpret.

In humans, the “gold standard” for assessing insulin resistance is the euglycemic hyperinsulinemic clamp (IS clamp) because it directly measures insulin action on glucose utilization under steady-state conditions. However, this technique is difficult and can only be used for a small number of subjects.^14^ There are a number of other more practical methods used in research and clinically larger-scale settings. The most popular measures, especially among health practitioners in Saudi Arabia, are the Homeostasis Model Assessment-Insulin Resistance (HOMA-IR)^15^ and the Quantitative Insulin Sensitivity Check Index (QUICKI),^16^ both being derived from fasting plasma glucose (FPG) and fasting plasma insulin (FPI) concentrations. Both correlate reasonably with the clamp technique;^16^,^17^ however, both have limitations.^17^–^19^ More recently, Perseghin et al,^20^ by incorporating fasting plasma free fatty acid (FFA) concentration into QUICKI {so that the modified QUICKI=1/[log(fasting insulin)+log(fasting blood glucose)+log(fasting FFA)]}, improved its correlation to the IS clamp and its discriminatory power in cases of mild insulin-resistant states.^21^ This method has never been tested on Saudi subjects. Furthermore, no validation tests for the use of any surrogate measures of insulin resistance in Saudi subjects using euglycemic clamp have ever been conducted, and it is not known whether they are able to identify insulin-resistant subjects using cut-off points published for other populations. Despite this, some reports on Saudi subjects used those cut-off points to diagnose IR.^8^,^22^ Moreover, practicing physicians working in Saudi Arabia are using them for diagnosis and management of overweight non-diabetic individuals.

The aim of this study was to employ this modified version (M-QUICKI), the original one, and the “Homeostasis Model Assessment of Insulin Resistance” (HOMA-IR) to separate selected non-diabetic adults into high- (HIR) and low-insulin-resistant (LIR) subgroups, using earlier reported cut-off points, in order to investigate whether this division produces similar results and whether it leads to a difference in well-known anthropometric and biochemical characteristics between LIR and HIR subgroups.

## METHODS

In a cross-sectional study, healthy subjects aged 18-50 years were recruited randomly from individuals visiting health centers during the period between July 2005 and January 2007. Six health centers (representing the six health sectors of Jeddah) were chosen randomly. Based on an earlier study of insulin resistance in Saudi diabetic individuals,^8^ the sample size to detect differences between means or medians of anthropometric measurements and estimated blood indices in HIR and LIR subgroups was computed using Power and Precision (Version 2) statistical analysis software (Biostat 2009, http://www.analystsoft.com/en/products/biostat/)and selecting the power of the study as 0.9 (90%). The calculated sample size was found to be 205. According to population density, a sample size of 209 was then calculated for each center, after rounding fractions. Exclusion criteria included the following: reported diabetes (or fasting plasma glucose ≥126 mg/dL, i.e., 7.0 mmol/L upon testing), endocrine disorders, hypertension, reported dyslipidemia and coronary heart diseases. Hypertension was defined as a systolic blood pressure >140 mm Hg or diastolic blood pressure >90 mm Hg^23^ or current use of antihypertensive medications. Dyslipidemia was defined as increased cholesterol level (total cholesterol level ≥5.2 mmol/L, an LDL-C ≥3.36 mmol/L and/or an HDL-C <1.04 mmol/L)^24^ and/ or increased level of triglycerides (≥1.7 mmol/L).^25^ Informed consent was obtained from all participants after explanation of purpose, nature and potential risks of the study. Ethical approval was granted by the bioethical and research committee. Recruits were checked for hypertension, and only normotensive individuals were interviewed for demographic information, and their anthropometric measurements were taken. Abdominal obesity was defined as >88 cm in females and >102 cm in males.^26^

Selected subjects were given an appointment for blood collection while fasting. Collected samples were immediately placed on ice prior to processing. Glucose was determined first in separated serum, and subjects showing hyperglycemia were excluded. Remaining samples were divided into aliquots and frozen at –70°C for later determination of lipid profile, insulin and FFA.

Glucose, total cholesterol, HDL-cholesterol, LDL-cholesterol and triglycerides (TGs) were estimated using automated enzymatic methods (Dade Behring Inc., UK). The coefficient of variation was <2% and <5% for intra- and inter-batch, respectively, in all cases. Insulin was estimated in one batch using the electro-chemiluminescence immunoassay (ECLIA) on Modular Analytics E 170 (Elecys module) immunoassay analyzer supplied by Roche Diagnostics GMbH, Germany. (CV was 9.7%.) All measurements were carried out at the university hospital biochemistry laboratory. FFAs were estimated manually in serum using an enzymatic method (Wako Chemicals GMbH, Germany, www.wako-chemicals.de), with intra- and inter-batch CV being 5.2% and 9.8%, respectively.

HOMA-IR, QUICKI and the modified QUICKI were calculated as reported earlier.^15^,^16^,^20^ Individuals whose samples had a value outside limits reported for non-insulin-resistant healthy subjects by Ascaso et al^27^ for HOMA-IR, Hrebicek et al^28^ for QUICKI and by Perseghin et al^20^,^21^ for modified QUICKI were labeled HIR. They were matched for age and sex to individuals from the rest of the study population, resulting in 3 HIR and 3 LIR subgroups.

Descriptive statistics such as mean (standard deviation) for normally distributed data or median and interquartile range (IQR) for non-normally distributed variables were calculated for all parameters in each of the six resulting subgroups. Statistical analyses were performed using the unpaired *t* test and Mann-Whitney U test for comparison of normally distributed and non-normally distributed parameters, respectively, while the Chi-square test was used to compare categorical parameters. A statistical computer program (SPSS) was used to analyze the data. Significance was assigned at *P*<.05.

## RESULTS

Three hundred fifty-seven subjects were recruited. Only 209 subjects (76 males and 133 females) satisfied the criteria and provided required samples. The demographic and anthropometric characteristics of the selected group are presented in Table 1, while their biochemical parameters are presented in Table 2.

**Table 1:** Demographic and anthropometric characteristics of the study group.

	Male	Female	Total
No. of subjects (%)	76 (36.4%)	133 (63.6%)	209 (100%)
Age (yrs)	33.0 (10.8)	31.3 (10.2)	31.8 (10.4)
Weight (kg)	73.2 (16.0)	67.2 (15.8)	69.3 (16.1)
Height (cm)	168.0 (9.5)	157.5 (7.6)	161.3 (9.7)
BMI (kg/m^2^)	25.7 (5.3)	26.90 (6.5)	26.44 (6.12)
BMI classes n (%):			
Normal (<25 kg/m^2^)	37 (48.7%)	59 (44.4%)	96 (46.0%)
Overweight (25-<30 kg/m^2^)	24 (31.6%)	35 (29.3%)	59 (28.2%)
Obese (≥30 kg/m^2^)	15 (19.7%)	39 (29.3%)	54 (25.8%)
Waist (cm)	87.2 (15.3)	82.3 (16.4)	84.6 (17.0)
Hip (cm)	98.4 (15.9)	105.1 (14.9)	103.3 (16.1)
Waist: Hip ratio	0.89 (0.015)	0.78 (0.08)	0.82 (0.15)
Family history of diabetes mellitus n (%)	39 (51.3%)	70 (52.6%)	109 (52.1%)

BMI: body mass index, n: number of subjects. Data are presented as mean±SD or number and percentage.

**Table 2:** Biochemical parameters of the study group.

	Male	Female	Total
No. of subjects (%)	76 (36.4%)	133 (63.6%)	209 (100%)
Total cholesterol (mmol/L)	5.1 (1.0)	5.2 (1.1)	5.2 (1.1)
TC ≥5.2 mmol/L, n (%)	30 (39.5%)	63 (47.4%)	93 (44.5%)
LDL- cholesterol (mmol/L)	3.28 (0.76)	3.02 (0.81)	3.11 (0.79)
LDL-cholesterol ≥3.36 mmol/L, n (%)	32 (42.1%)	49 (36.8%)	81 (38.8%)
HDL-cholesterol (mmol/L)	1.25 (0.29)	1.58 (0.39)	1.46 (0.39)
HDL-cholesterol <1.09 mmol/L, n (%)	20 (26.3%)	13 (9.8%)	33 (15.8%)
TG (mmol/L)	1.32 (1.00-1.86)	0.99 (0.76-1.45)	1.1 (0.80-1.6)
Glucose (mmol/L)	5.6 (0.80)	5.5 (0.80)	5.5 (0.80)
Insulin (mU/L)	7.5 (4.4-14.3)	7.8 (5.7-11.1)	7.7 (5.3-11.5)
FFA (mg/dL)	8.0 (5.3-10.8)	8.8 (6.1-11.6)	8.4 (5.8-11.3)

FFA: free fatty acids, TC: total cholesterol, TG: triglycerides, n: number of subjects. Data are presented as mean (SD) for normally distributed parameters and as median and interquartile range for non-normally distributed ones.

Using the QUICKI and a cut-off point of <0.357,^28^ 135 subjects (64.6% of the studied population) were considered to have high insulin resistance. Of these, 113 subjects (87.7%), were either obese or overweight; and 73 (54.1%) suffered from abdominal obesity (>102 and >88 cm for males and females, respectively). In contrast, subjects in the LIR subgroup had normal BMI, and none suffered from abdominal obesity.

The division between sexes in the HIR subgroup was not significantly different from that in the group as a whole (*P*=.76) since it included 47 males (34.8%) and 88 females (65.2%). Due to limitations of sample size, matching was not done on a one-to-one basis, but was done keeping in mind age and the ratio of the sexes. This resulted in exclusion of some of the HIR subjects, giving a total number of 121 subjects in the HIR subgroup (42 males and 79 females) and 74 subjects in the LIR subgroup (29 males and 45 females). The calculated age means (SD) did not differ significantly between the two subgroups (31.9 [10.3] for HIR subgroup and 31.7 [10.5] for LIR subgroup, *P*=.43). However; when the Chi-square test was applied, the distribution between the different classes of BMI was significantly different in the two subgroups (*P*<.01), and the percentage of subjects with abdominal obesity was also significantly higher in the HIR subgroup (*P*=.047). On the other hand, more than 50% of the subjects in both subgroups reported a family history of diabetes, with no significant difference in the percentage between the two subgroups (*P*=.67).

The HIR subgroup did not have most of the well-recognized biochemical characteristics of insulin resistance. Its calculated means or medians of measured parameters did not significantly differ from the corresponding means or medians of the LIR subgroup determined by the same index (results not shown). Most had a normal lipid profile, with total cholesterol level being ≥5.2 mmol/L in 42 individuals (34.7% of the entire subgroup) compared to 39.1% in the LIR subgroup. LDL-cholesterol level was ≥3.36 mmol/L in 38 subjects (31.4%) compared to 40.5% in the LIR subgroup, and HDL-cholesterol was ≤1.09 mmol/L in 18 subjects (14.9%) compared to 16.2% in the LIR subgroup. Furthermore, only 20 subjects (16.5% of the HIR subgroup) had higher-than-acceptable plasma triglycerides (i.e., ≥1.7 mmol/L) compared to 18.9% in the LIR subgroup. Moreover, the majority of subjects in the HIR subgroup (80 individuals, or 66.1%) had a glucose value <6.0 mmol/L and/or insulin and FFA values in the lowest quartile of the calculated range for the studied population.

Using the modified QUICKI and a cut-off point of <0.419,^20^,^21^ 97 individuals (46.4% of the total) were identified as having HIR, including 34 males (35.1%) and 63 females (64.9%), with 84 of them being also identified by QUICKI. The division between sexes was not significantly different from that in the group as a whole (*P*=.76).

Matching for age and sex could be done for 90 IR subjects only. The HIR group had significantly higher mean weight, higher percentage of obese individuals and higher percentage of subjects with abdominal obesity (Table 3). In addition, means or medians of all estimated biochemical parameters (except HDL-cholesterol) were significantly higher in the HIR group (Table 4). Modified QUICKI for all subjects correlated significantly (*P*=.01) with HOMA values (r =–0.756) and with QUICKI values (r=0.758).

**Table 3:** Anthropometric and demographic characteristics of high–insulin-resistant (HIR) and low–insulin-resistant (LIR) subgroups using modified QUICKI.

	HIR (n=90)	LIR (n=90)	*P*
Age (years)	32.4 (9.9)	31.7 (9.5)	.66
Weight (kg)	74.1 (17.9)	66.5 (14.0)	.002
BMI (kg/m^2^)	29.0 (7.1)	25.6 (4.6)	0
BMI classes n (%):			
Normal (<25 kg/m^2^)	26 (28.89%)	41 (45.6 %)	
Overweight (25-29.9 kg/m^2^)	25 (27.78%)	34 (37.8 %)	4.29×10^-3^
Obese (≥30 kg/m^2^)	38 (42.22%)	15 (16.7 %)	
Waist (cm)	88.5 (16.7)	82.2 (14.8)	.008
Hip (cm)	105.7 (15.9)	99.3 (14.5)	.006
Waist: Hip ratio	0.84 (0.10)	0.83 (0.11)	.68
Waist >88 cm (F) or>102 cm (M), n (%)	37 (41.1%)	19 (21.1%)	4.3×10^-3^
Family history of diabetes mellitus n (%)	48 (53.3%)	51 (56.7%)	.69

BMI: body mass index, n: number of subjects. Continuous variables were compared by *t* test for normally distributed and Mann-Whitney *U* test was used for non-normally distributed parameters. Categorical data were compared by χ^2^ test.

**Table 4:** Biochemical parameters of high–insulin-resistant (HIR) and low–insulinresistant (LIR) subgroups using modified QUICKI.

	HIR (n=90)	LIR (n=90)	*P*
TC (mmol/L)	5.5 (1.1)	5.0 (1.0)	.003
TC ≥5.2 mmol/L, n (%)	51 (56.6%)	31 (34.4%)	9.14×10^-6^
LDL- cholesterol (mmol/L)	3.3 (0.8)	2.97 (0.78)	.008
LDL-cholesterol ≥ 3.36 mmol/L, n (%)	45 (50%)	29 (32.2%)	3.1×10^-4^
HDL-cholesterol (mmol/L)	1.5 (0.4)	1.5 (0.4)	1
HDL-cholesterol <1.09 mmol/L, n (%)	13 (14.4%)	14 (15.6%)	.77
TG (mmol/L)	1.3 (0.9 -1.9)	1 (0.8-1.4)	.002
TG ≥ 1.7 mmol/L, n (%)	17 (18.9%)	0 (0%)	0
Glucose (mmol/L)	5.7 (0.8)	5.4 (0.7)	.025
Insulin (mU/L)	11.2 (8.3-14.2)	6.1 (4.2-8.4)	0
FFA (mg/dL)	10.7 (8.4-13.1)	6.5 (4.8-9.3)	0

FFA: free fatty acids, n: number of subjects, TC: total cholesterol, TG: triglycerides. Continuous variables were compared by t test, and Mann-Whitney *U* test was used for comparison of normally distributed and non-normally distributed parameters. Categorical data were compared by χ^2^ test.

Using the HOMA index and a cut-off point of >3.8,^27^ only 26 subjects were identified as having HIR, representing 12.4% of the study population. They included 9 males and 17 females, representing 34.6% and 65.4% of the HIR subgroup, respectively. This division between sexes was not significantly different from that in the group as a whole (*P*=.79). Twenty-three of these subjects were also identified by modified QUICKI to have HIR. All of these individuals had the well-recognized characteristics of IR as diagnosed by the IS clamp technique, such as obesity (BMI ≥30 kg/m^2^) and abdominal obesity. All of them had a family history of diabetes. Twenty-two of them (84.6%) had high total and LDL-cholesterol, fasting glucose >6.0 to <7.0 mmol/L, and insulin, TG and FFA values in the upper quartile of the calculated range for the studied population. When means or medians of anthropometric and biochemical characteristics of the HIR and LIR subgroups divided according to this index and matched for age and sex (results not shown) were compared statistically and significant differences (*P*<.001) were found in all cases.

Only 22 subjects were identified by all the three indices. These were all obese, with abdominal obesity, abnormalities in lipid profile, blood glucose >6.0 mmol/L, insulin and FFA levels in the upper quartile of the calculated range for the studied population.

## DISCUSSION

Approximately 42% of the randomly recruited population had blood glucose values ≥7 mmol/L and had to be excluded according to our criteria. Therefore, the prevalence of diabetes in our region of the country could be much more than reported. Screening for insulin resistance and management of the condition would help prevent, or at least delay, the onset of diabetes.

Our calculated percentages for overweight and obese individuals in both sexes were lower than those reported earlier by Alsaif et al.^29^ This was most likely due to our exclusion criteria, but could also be due to differences in geographical location in the country between the two studies. In spite of this lower percentage, the fact that more than half of the population is overweight or obese is alarming. Obesity is a critical health care concern because of the vast associated burden of premature morbidity and mortality. Obesity contributes to hypertension, high serum cholesterol, low HDL-cholesterol and hyperglycemia, and it is otherwise associated with higher CVD risk. It is therefore unsurprising to find that approximately 45% of the studied population had unacceptably high total cholesterol value (≥5.2 mmol/L). This is more than the percentage reported by Al-Nuaim.^30^ Moreover, a slightly higher percentage of females were considered to be hypercholesterolemic compared to males, which could be explained by much higher mean HDL-cholesterol in females (*P*=.001). The high percentage of hypercholesterolemia places our population at high risk of cardiovascular disease, with the males having a higher risk considering their higher LDL and lower HDL levels and the fact that the median triacylglycerols value was significantly higher (*P*=.021).

Our use of different indices to identify individuals with HIR in our population gave widely differing results. Despite the fact that HOMA-IR and QUICKI both use fasting insulin and glucose levels to calculate insulin resistance and both correlate reasonably with the clamp technique,^16^,^17^ their results did not concur, with HOMA-IR identifying 12.4% of the study population as HIR compared to 64.6% being identified using QUICKI. One explanation for this is that cut-off levels employed in our study were developed for other populations and might be unsuitable for the studied group, which included a high percentage of overweight and obese individuals. However, a well-known limitation of HOMA-IR is its low sensitivity for detecting insulin resistance in mildly resistant cases or in lean individuals with beta cell dysfunction,^17^,^31^ which might offer an explanation for the much lower percentage identified by this index.

Another reason for the suspected underestimation by HOMA could be the fact that this method reflects hepatic IR only, not IR at peripheral tissues.^32^ Our studied population included subjects that could be pre-diabetic with an FPG concentration between 100 and 125 mg/dL (5.6-6.9 mmol/L) and referred to as having IFG,^33^ as well as subjects that could have abnormal postprandial glucose excretion but normal FPG concentration and referred to as having impaired glucose tolerance (IGT).^33^ Some subjects could also have a combination of IGT and IFG. Subjects with IFG have hepatic IR and thus can be detected by HOMA-IR. However, subjects with IGT have peripheral IR and hence will be missed.^32^

Theoretically, the above could also be applied to QUICKI, and subjects with IGT are expected to be missed. However, compared to HOMA-IR, QUICKI is reported to have the advantage of being applicable to wider ranges of insulin sensitivity,^31^,^34^ which might explain the much higher percentage labeled as HIR. But unlike what was noted when HOMA-IR was used, the HIR subgroup identified by QUICKI did not have most of the well-recognized biochemical characteristics of insulin resistance, and the calculated means or medians of their measured parameters did not significantly differ from those of the LIR subgroup. Moreover, the majority of subjects in the HIR subgroup (80 individuals, or 66.1%) had a glucose value <6.0 mmol/L, which does not conform to the WHO definition for insulin resistance.^25^ Thus it can be suggested that there might be an overestimation of insulin resistance when QUICKI with reported cut-off values is used in Saudi subjects, as reported earlier for other populations.^35^ Another possibility is that QUICKI identifies HIR individuals before the appearance of biochemical abnormalities, which could take some time to develop.

On the other hand, using the modified QUICKI and the cut-off point reported by Perseghin et al^20^ (i.e., 0.419) for non-diabetic subjects, 97 individuals (46.4% of total) were identified as having HIR, with 84 of them being also identified by QUICKI. The HIR subgroup had significantly higher mean weight, mean BMI, mean waist and hip circumferences compared to the LIR subgroup, and a higher percentage of HIR subjects suffered from abdominal obesity.

Insulin resistance in adipose tissue will lead to elevated plasma FFA. Therefore, inclusion of FFA into the QUICKI formula can be beneficial and can increase its detection power by including those subjects with peripheral IR, especially in view of the following: (1) increased fasting FFA concentration could reflect insulin resistance earlier than hyperglycemia since lipolysis is more sensitive to insulin than glucose utilization;^36^ (2) a small increase in plasma FFA concentration in healthy individuals is reported to induce insulin resistance,^37^ and (3) insulin resistance of lipolysis was suggested as explaining about 10% of the variation in insulin sensitivity of glucose disposal (4) in normal subjects;^36^ (5) dysfunctional regulation of lipolysis was established in insulin-resistant subjects.^38^ In fact, modified QUICKI has already been reported to be better correlated with clamp measurement than the original QUICKI or HOMA-IR.^20^,^21^

The superior ability of modified QUICKI to identify HIR individuals in Saudi subjects is also strongly suggested by the finding that the means or medians of almost all biochemical parameters, except HDL-cholesterol, were significantly higher in the LIR subgroup. Thus, the identified HIR subgroup using this index had all the well-recognized characteristics of insulin resistance as diagnosed by the gold standard method, unlike that identified using QUICKI.

Even though it is difficult with the available data to decide which of the three indices gives a better estimation of insulin resistance in Saudi non-diabetic normotensive individuals, our data show that when the three indices were used to identify HIR individuals, division between sexes was not significantly different from that in the group as a whole. Therefore, it can be suggested that sex is not a risk factor for insulin resistance in the studied population.

Furthermore, increased body fat content was noted when all three indices were used to identify HIR individuals, as reported earlier.^39^ Excess adipose tissue releases several products that apparently exacerbate these risk factors. They include nonesterified fatty acids (NEFA), cytokines, PAI-1 and adiponectin. A high plasma NEFA level overloads muscle and liver with lipid, which enhances insulin resistance.^40^ All subjects identified by HOMA were obese and showed abdominal obesity, while 88% of HIR subjects identified by QUICKI were either overweight or obese, and more than half had abdominal obesity. Abdominal obesity, in particular, correlates with metabolic risk factors.^41^ Even though the HIR group, using modified QUICKI, had significantly higher mean weight, mean BMI, mean waist and hip circumferences (Table 3) compared to LIR group, and a higher percentage of HIR subjects suffered from abdominal obesity, a higher percentage of subjects with normal BMI and no abdominal obesity were included. A broad range of insulin sensitivities exists at any given level of body fat.^39^ Most people with categorical obesity (body mass index [BMI], ≥30 kg/m^2^) have postprandial hyperinsulinemia and relatively low insulin sensitivity,^41^ but variation in insulin sensitivities exists even within the obese and overweight (BMI, 25-29.9 kg/m^2^) populations, suggesting an inherited component to insulin resistance.^39^ In some populations (e.g., south Asians), insulin resistance occurs commonly even with BMI <25 kg/m^2^ and apparently contributes to a high prevalence of type 2 diabetes and premature CVD.^40^ Individuals who manifest insulin resistance with no or only mild-to-moderate overweight can be said to have primary insulin resistance. Furthermore, it was reported that weight gain seems to enhance insulin resistance and metabolic syndrome in case of primary insulin resistance.^40^ Thus, it can be suggested that individuals with normal weight, identified by QUICKI or modified QUICKI in our studied population to have HIR, might have primary insulin resistance, especially that they included subjects of south Asian ethnicity (ethnicity data are not shown). On the other hand, in view of the absence of normal-weight individuals in the HIR subgroup identified by HOMA, it might be suggested that this index identifies categorical insulin resistance but misses primary insulin resistance. Even though correlation analysis with the clamp technique was not conducted in this work, important data was collected and some tentative conclusions can be made. First of all, overweight and obesity is associated with high insulin resistance in our population; however, high insulin resistance might be found also in individuals with normal body fat content and weight. Secondly, sex does not appear to be a risk factor in the studied population. Thirdly, none of the three indices, with the cut-off points determined earlier for other populations, can be used solely to identify insulin-resistant subjects in a clinical setting for treatment purposes. Finally, the significant difference found when modified QUICKI was employed, between the HIR and the LIR subgroups in anthropometric and biochemical characteristics might justify the additional cost of estimating FFA in future surveys to diagnose insulin resistance in our Saudi population. However, since only 22 subjects were identified by all three indices to have HIR, more studies using the clamp technique are needed to clarify discrepancies in results and to determine the correct cut-off points for diagnosing insulin resistance in different population subgroups according to their BMI or class of obesity. Future work should also look at including other measures of lipolysis, such as glycerol.
